# Effect of Mesenchymal Precursor Cells on the Systemic Inflammatory Response and Endothelial Dysfunction in an Ovine Model of Collagen-Induced Arthritis

**DOI:** 10.1371/journal.pone.0124144

**Published:** 2015-05-07

**Authors:** Laura M. Dooley, Anwar Abdalmula, Elizabeth A. Washington, Claire Kaufman, Elizabeth M. Tudor, Peter Ghosh, Silviu Itescu, Wayne G. Kimpton, Simon R. Bailey

**Affiliations:** 1 Faculty of Veterinary Science, The University of Melbourne, Parkville, Victoria, Australia; 2 Mesoblast Ltd, Melbourne, Victoria, Australia; Wake Forest Institute for Regenerative Medicine, UNITED STATES

## Abstract

**Background and Aim:**

Mesenchymal precursor cells (MPC) are reported to possess immunomodulatory properties that may prove beneficial in autoimmune and other inflammatory conditions. However, their mechanism of action is poorly understood. A collagen-induced arthritis model has been previously developed which demonstrates local joint inflammation and systemic inflammatory changes. These include not only increased levels of inflammatory markers, but also vascular endothelial cell dysfunction, characterised by reduced endothelium-dependent vasodilation. This study aimed to characterise the changes in systemic inflammatory markers and endothelial function following the intravenous administration of MPC, in the ovine model.

**Methods:**

Arthritis was induced in sixteen adult sheep by administration of bovine type II collagen into the hock joint following initial sensitisation. After 24h, sheep were administered either 150 million allogeneic ovine MPCs intravenously, or saline only. Fibrinogen and serum amyloid-A were measured in plasma to assess systemic inflammation, along with pro-inflammatory and anti-inflammatory cytokines. Animals were necropsied two weeks following arthritis induction. Coronary and digital arterial segments were mounted in a Mulvaney-Halpern wire myograph. The relaxant response to endothelium-dependent and endothelium-independent vasodilators was used to assess endothelial dysfunction.

**Results and Conclusion:**

Arthritic sheep treated with MPC demonstrated a marked spike in plasma IL-10, 24h following MPC administration. They also showed significantly reduced plasma levels of the inflammatory markers, fibrinogen and serum amyloid A, and increased HDL. Coronary arteries from RA sheep treated with MPCs demonstrated a significantly greater maximal relaxation to bradykinin when compared to untreated RA sheep (253.6 ± 17.1% of pre-contracted tone vs. 182.3 ± 27.3% in controls), and digital arteries also demonstrated greater endothelium-dependent vasodilation. This study demonstrated that MPCs given intravenously are able to attenuate systemic inflammatory changes associated with a monoarthritis, including the development of endothelial dysfunction.

## Introduction

Mesenchymal stem cells (MSCs) are a type of adult stem cell able to differentiate into cells of the mesodermal lineage [1,2]. In addition to their potential regenerative capability, MSCs have also been found to possess broad immunomodulatory properties; they are capable of interacting with a range of immune effector cells to induce peripheral tolerance and exert an anti-inflammatory effect [3]. Mesenchymal precursor cells (MPC) are a subset population of MSCs which are STRO-1^bright^ STRO-3^bright^ and exhibit the capacity for multi-differentiation and extensive proliferation [4]. MPC have been used allogeneically in animal models and in human trials; including applications such as acute myocardial infarction and intervertebral disc disease [5,6,7]. MPC hold promise for the development of an ‘off the shelf’ stem cell product which could be stored on site and administered intravenously when needed. The anti-inflammatory mechanism of action of MSCs is poorly understood; in particular, the interactions between MSC and immune cells is the subject of current research. Important mediators may include PGE_2_ and indoleamine which are thought to modulate T cells, monocytes and dendritic cells [3].

Many chronic inflammatory conditions, such as rheumatoid arthritis (RA), obesity and diabetes, are associated with a low-grade systemic inflammatory response which affects many body systems directly and indirectly; particularly the cardiovascular system. For example, in RA patients, cardiovascular diseases represent an important co-morbidity and contribute to an increased mortality rate amongst this patient population [8,9,10]. Systemic inflammation contributes to the initiation and development of cardiovascular diseases through a number of mechanisms, including direct actions of circulating mediators on endothelial cells, and modulation of lipid phenotype [11,12].

Endothelial dysfunction is a general term describing an abnormal response of the endothelium to physiological stimuli, and may be characterised by reduced endothelium-dependent vasodilation [13]. This is thought to be due primarily to a reduction in the bioavailability of the important endothelium-derived vasodilator nitric oxide (NO). Endothelial dysfunction is thought to be one of the earliest detectable cardiovascular changes in the development of atherosclerosis, and is assessed clinically in human patients by flow-mediated dilation of the brachial artery [14,15,16]. Endothelial dysfunction has been linked to an increased risk of the development of clinical cardiovascular disease in the short and long term [17,18,19,20].

Previous studies by our group have demonstrated that in an ovine model of collagen-induced arthritis (CIA) involving just the left hock joint, there is evidence of systemic inflammation that also produces endothelial dysfunction in both the coronary and peripheral vascular bed [21,22]. This model therefore provides an opportunity to study the earliest cardiovascular changes associated with mild systemic inflammation. It was hypothesised that the immunomodulatory actions of MPC, which target multiple immune and inflammatory mechanisms, may provide superior therapeutic effects to current biological therapies which target only a very specific part of the inflammatory response (for example, anti-TNF-α medications). Additionally, it is proposed that these cells will also have favourable cardiovascular effects in systemic inflammatory diseases through a reduction in inflammation-driven endothelial dysfunction.

This study aimed to assess the effect of allogeneic bone marrow derived ovine MPCs, administered intravenously, on circulating inflammatory mediators and the development of endothelial dysfunction in a previously validated ovine model of rheumatoid arthritis. The specific hypothesis for this study was that intravenous MPCs would prevent or reduce the development of systemic inflammation associated with arthritis and also prevent the subsequent vascular endothelial dysfunction in the heart and peripheral circulation.

## Methods

### Animals

Sixteen merino ewes, 12–18 months of age were obtained from a local supplier. They were housed in mesh floor pens and given access to pelleted food and water ad libitum. Sheep were acclimatized to housing for 2 weeks before the commencement of experiments, during which time they underwent a clinical veterinary examination. Blood samples were taken to ensure that all animals were free of any inflammatory conditions that may have impacted the results of the study. Sheep were allocated randomly into groups. All procedures were carried out with the permission of the Melbourne University Animal Ethics Committee (ID 1212422.2).

### Induction of arthritis

Bovine type II collagen solutions were prepared as previously described [23]. Arthritis was induced using a 6 week protocol, as previously described [21,22]. Sheep initially received a subcutaneous injection of bovine type II collagen solution (5mg/mL) in Freund’s complete adjuvant. Two weeks later, the sheep received another subcutaneous injection of bovine type II collagen (5mg/mL) in Freund’s incomplete adjuvant. Two weeks following this second subcutaneous injection, 100 ug of bovine type II collagen in sterile phosphate buffered saline (total volume 0.5mL) was administered into the left tibiotarsal joint, at which time clinical arthritis is induced (day 0). Sheep were killed and necropsied two weeks following their intra-articular injection. Euthanasia was performed using a lethal dose of intravenous pentobarbitone (Lethabarb, Virbac, Australia).

### Mesenchymal Precursor Cell treatment

Allogeneic ovine mesenchymal precursor cells (MPC) were provided by Mesoblast Ltd. The cells were cryopreserved in ampoules containing 30 million MPC/mL of a solution comprising ProFreeze, DMSO and modified Eagle’s medium (αMEM). MPC treatment was administered to the sheep 24h following arthritis induction (day 1). Ampoules were thawed rapidly, and cell number and viability assessed using trypan blue staining and a Neubauer haemocytometer. The appropriate volume for a dose of 150 million viable MPCs was calculated and injected (using an 18g needle) into a sterile 100mL bag of 0.9% sodium chloride connected to a giving and extension set including a 40/150 micron dual screen filter to trap any cell clumps. This was delivered as a constant rate infusion using a fluid pump over a period of 30 minutes. Treatments were administered intravenously via a 14G catheter placed in the jugular vein. Control sheep received an equivalent volume of sterile saline delivered in an identical manner via injection into a 100mL bag of 0.9% Sodium Chloride. Investigators involved in data collection remained blinded to treatment group allocations for the duration of the study. The dose of 150 million MPC was derived from previous studies in sheep conducted in our laboratory [24].

### Blood sampling and measurement of inflammatory mediators

Blood was collected from the jugular vein on the day of arthritis induction (day 0, immediately prior to the intra-articular injection), then days 1, 2, 3, 4, 6, 8, 9, and 14 (the day of euthanasia). Plasma fibrinogen was measured using the modified heat precipitation method at a commercial veterinary laboratory (Australian Specialised Animal Laboratories, Australia). Serum samples were also submitted to a commercial veterinary laboratory for measurement of high density lipoprotein (HDL) and low density lipoprotein (LDL) (Gribbles Pathology, Australia). Serum amyloid-A (SAA) was measured using a commercial ELISA according to manufacturer’s instructions (Multispecies SAA ELISA kit, Tridelta Development Limited, Ireland).

The concentrations of IL-6, IL-10, and TNF-α were measured by sandwich ELISA. IL-6 was estimated using anti-ovine IL-6 (4B6, AbD Serotec) and rabbit anti-ovine IL-6 (AHP424, AbD Serotec) followed by goat anti-rabbit-HRP (Life Technologies, Waltham, MA, USA). The standard was recombinant ovine IL-6 (RP0014B, Kingfisher Biotech, St Paul, MN, USA). IL-10 was estimated using anti-bovine IL-10 (cc318, AbD Serotec) and biotinylated anti-bovine IL-10 (cc320, AbD Serotec) followed by ExtrAvidin-HRP (Sigma-Aldrich). The standard was recombinant ovine IL-10, produced as described previously [25]. TNF-α was estimated using anti-ovine TNF-α (6.06, obtained from A/Prof. Scheerlinck, Centre for Animal Biotechnology, The University of Melbourne, Australia) [26] and biotinylated anti-bovine TNF-α (cc328, AbD Serotec) followed by ExtrAvidin-HRP (Sigma-Aldrich). The standard was recombinant bovine TNF-α (2279-BT, R&D Systems, Minneapolis, MN, USA). The intra-assay coefficients of variation for these assays were <6% and the inter-assay coefficients of variation were <19%.

### Wire Myography: Arterial Preparation

Arterial segments were prepared as described previously [22]. Following euthanasia, tissues were collected and placed in ice-cold oxygenated modified Krebs-Henseleit solution (KHS, 118.0mM/L NaCl, 4.7mM/L KCl, 1.2 mM/L MgSO_4_, 1.2mM/L KH_2_PO_4_, 25.0 mM/L NaHCO_3_, 11.1mM/L D-glucose and 2.5mM/L CaCl_2_). A dissecting microscope (Olympus SZ61, Olympus Australia) was used to identify a second order branch of the left descending coronary artery and dissect it free from surrounding tissue. Digital artery segments were collected from the first branch of the palmar digital artery from the left forelimb. Arteries were cut into 1–2mm segments and mounted on 40 μm wires in a Mulvaney-Halpern wire myograph (Danish Myo Technologies, Denmark) coupled to a data acquisition system (Power Lab, ADI Instruments, Oxfordshire, UK) to enable isometric tension recording. This apparatus is used to measure the force generated by small blood vessels maintained under physiological tension in a bath of Krebs-Henseleit solution. The segment of blood vessel (up to 2mm long) is restrained on wires, one of which is connected to a force transducer and the other to a variable micrometer. Once the blood vessel is equilibrated and tension is normalized to a point of passive stretch suitable for the development of active tension, it can be contracted with suitable vasoconstrictor mediators in a dose-dependent manner, and then vasorelaxation may be subsequently induced.

Arteries were maintained in oxygenated (95%O_2_ and 5%CO_2_) KHS at 37°C. Preparations were allowed to equilibrate for 30 minutes before a normalization procedure was performed to determine the optimal internal circumference. The viability of each vessel segment was assessed initially by measurement of the contractile response to standard depolarizing Krebs solution (DKS; 118mM KCl).

### Wire Myography: Coronary arteries

Two coronary artery segments were mounted for each individual animal. Segments were contracted with endothelin-1 (10^–8^ M; Sigma-Aldrich). Indomethacin (10^-5^M) was added to the KHS solution. Once the contractile tension had reached a plateau, one vessel segment from each individual was dilated with the endothelium-dependent vasodilator, bradykinin (10^-11^M - 3x10^-6^M; Sigma-Aldrich, Australia) and another segment dilated with sodium nitroprusside (SNP, 10^-9^M - 3x10^-4^M; Sigma-Aldrich). Arterial responses were recorded using Chart software (version 5.0).

### Wire Myography- Digital arteries

Two digital artery segments were mounted for each individual animal. Segments were contracted with incremental doses of 5-hydroxytryptamine (5HT; 10^-8^M - 10^-4^M; Sigma Aldrich, Australia) to establish maximal arterial contraction. This was followed by a wash-out period, in which vessels were allowed to re-equilibrate to resting tension. 5HT was then added at a concentration appropriate to produce a contraction of approximately 75% of maximal contraction for the individual arterial segment. Once the contractile tension had reached a plateau, one vessel segment from each individual was dilated with the endothelium-dependent vasodilator, carbachol (10^-8^M - 10^-3^M; Sigma-Aldrich) and another segment dilated with SNP (10^-9^M - 3x10^-4^M; Sigma-Aldrich).

### Data analysis

Myograph data is presented as cumulative concentration response curves, with results expressed as mean ± SEM. For each experiment *n* indicates the number of individual sheep used. A curve-fitting program (Graph Pad Prism, Version 6.02) was used to calculate the maximal response value for each individual curve. The equation used to fit the dose response curves was: E = [E_max_ A^nH^/ (A^nH^ + EC_50_)]. Emax is the maximum response and nH represents the Hill slope.

The maximal response values of the two groups were compared using an unpaired t-test, with significance accepted at p ≤ 0.05. Inflammatory markers measured in plasma over days 0–14 following arthritis induction are presented as mean ± SEM at each time point. The statistical program Graph Pad Prism (Version 6.02) was used to compare the two groups using a 2 way ANOVA with Bonferroni’s post-hoc test (SAA, Fibrinogen, HDL) or Sidak’s multiple comparison test (IL-10, IL-6). Normality was determined using the Shapiro-Wilk test.

## Results

### Pro- and anti-inflammatory mediators

**Fig 1:**
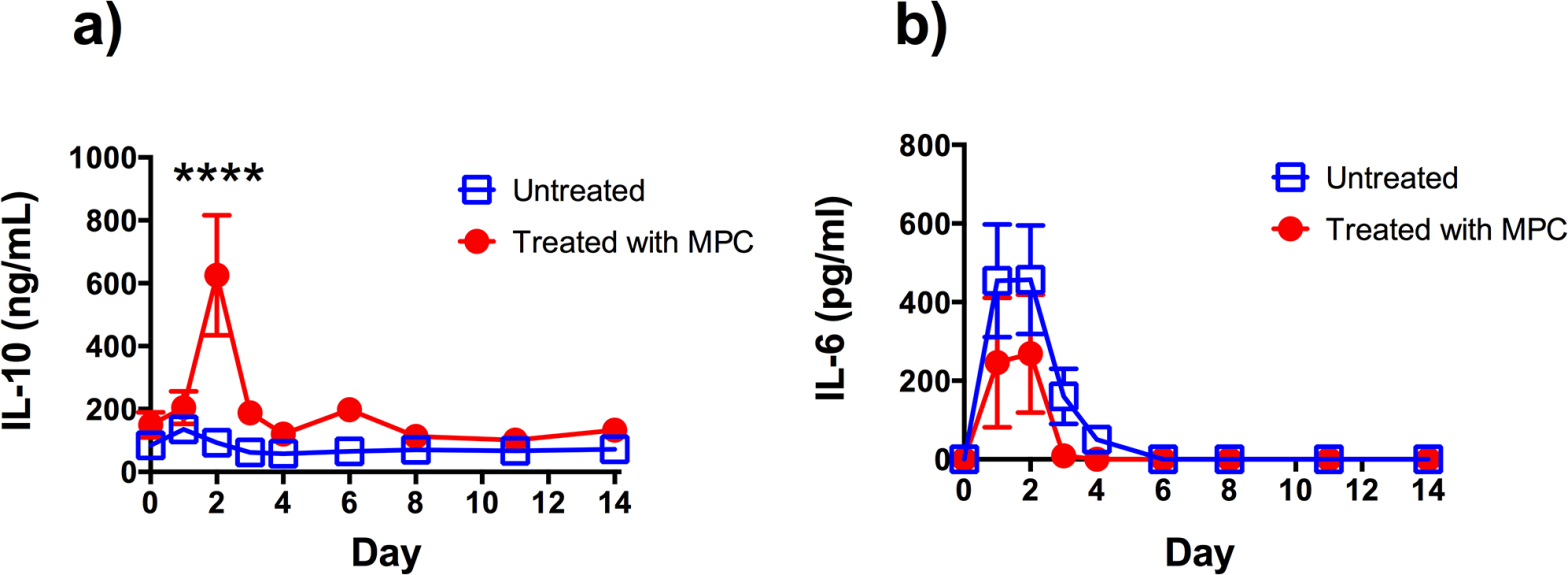
Comparison of plasma concentrations of (A) IL-10 and (B) IL-6 measured over time in arthritic sheep treated and untreated with mesenchymal precursor cells (MPC). Each point represents the mean ± SEM of 7–8 sheep.**** Indicates a statistically significant difference between groups at that time point, p ≤ 0.0001.

The most striking effect of MPC treatment on plasma cytokine levels was in the response of anti-inflammatory IL-10. There was a significant spike in plasma IL-10 of sheep treated with MPC, one day after MPC treatment, where it reached a mean concentration of over 600 ng/ml (Fig 1A). This peak was absent in the saline treated sheep. IL-6 levels in plasma peaked in the two days following IA collagen administration (Fig1B). Although levels became undetectable in the treated group by day 31, earlier than the non-treated animals (day 34), there was considerable variability and no significant difference was found between the MPC treated and saline control groups. Levels of TNF-α were very low and close to the limit of detection of the assay. No significant differences were observed between the two groups.

**Fig 2:**
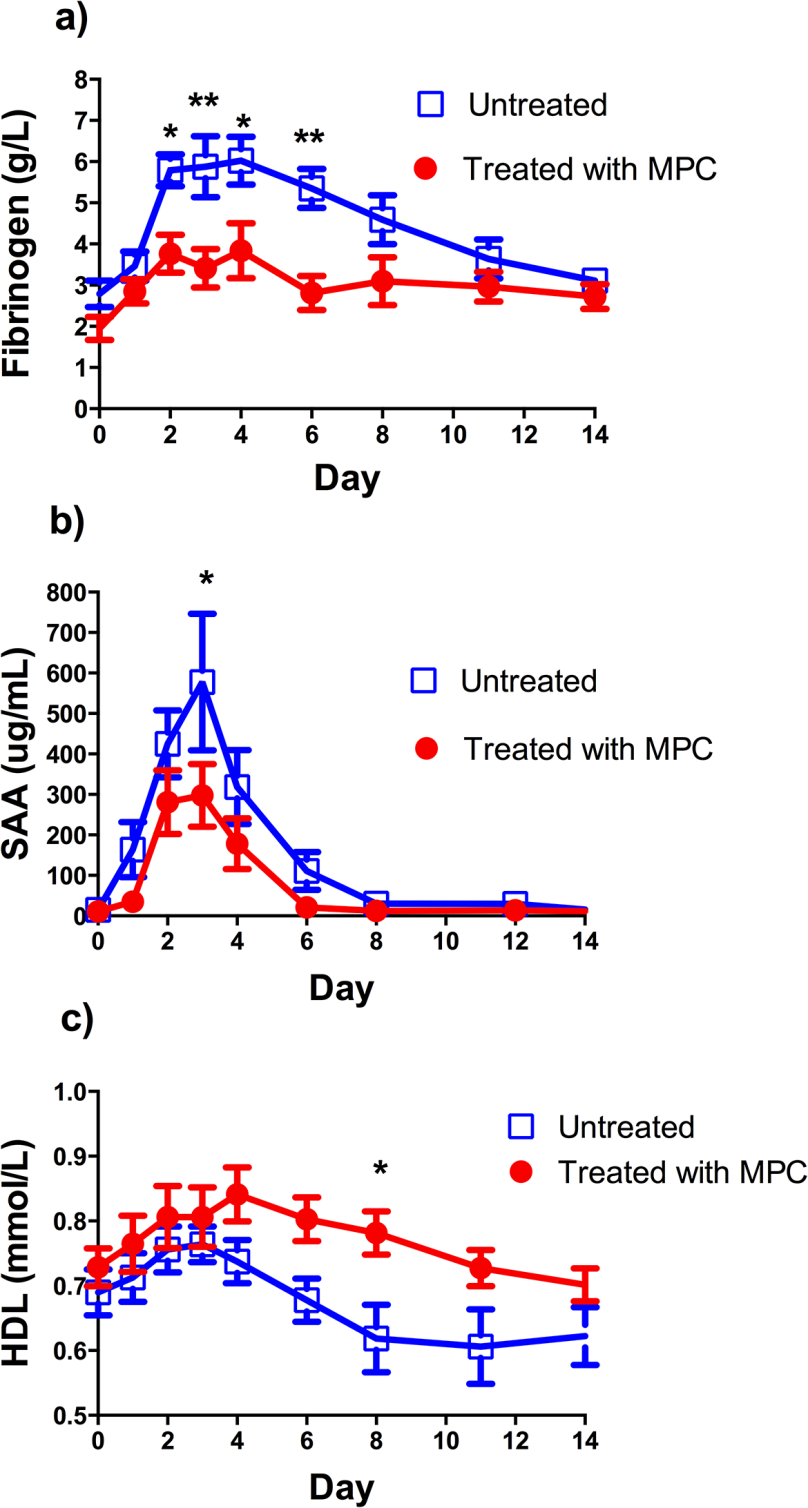
Comparison of plasma concentrations of (A) Fibrinogen, (B) Serum amyloid-A (SAA), (C) High density lipoprotein (HDL) measured over time in arthritic sheep treated and untreated with mesenchymal precursor cells (MPC). Each point represents the mean ±SEM from 8 animals. * Indicates a statistically significant difference between groups at that time point, p< 0.05, ** indicates a statistically significant difference between groups at that time point, p< 0.01.

Following the induction of arthritis (day 0), all sheep showed an increase in plasma fibrinogen consistent with a systemic inflammatory response (Fig 2A). Arthritic sheep treated with 150 million allogenic MPC on day 1 following arthritis induction demonstrated significantly lower plasma fibrinogen levels on days 2 (mean of 3.8 vs 5.8g/L for treated and untreated sheep respectively), 3 (mean of 3.4 vs 5.9g/L treated and untreated sheep respectively), 4 (mean of 3.8 vs 6.0g/L for treated and untreated sheep respectively) and 6 (mean of 2.8 vs 5.3g/L for treated and untreated sheep respectively) following arthritis induction.

All sheep showed a rapid increase in circulating serum amyloid-A over the first 4 days following arthritis induction, which waned by day 6–8. Arthritic sheep treated with 150 million allogenic MPCs on day 1 following arthritis induction demonstrated significantly lower serum amyloid-A levels on day 3 following arthritis induction (mean of 297.8 vs 577.7ug/mL for treated and untreated sheep respectively, Fig 2B).

Following arthritis induction, most sheep displayed a small increase in serum HDL over the first 3–4 days before displaying a gradual decline in HDL over the subsequent 10 days (Fig 2C). At day 8 following arthritis induction, the arthritic sheep treated with 150 million MPC intravenously showed a significantly higher plasma HDL than arthritic sheep treated with saline only (mean of 0.8 vs 0.6mmol/L for treated and untreated sheep respectively, Fig 2C). Serum LDL levels showed an approximately inverse trend to HDL levels, however there was no significant difference between the two groups (data not shown).

### Coronary arteries

**Fig 3:**
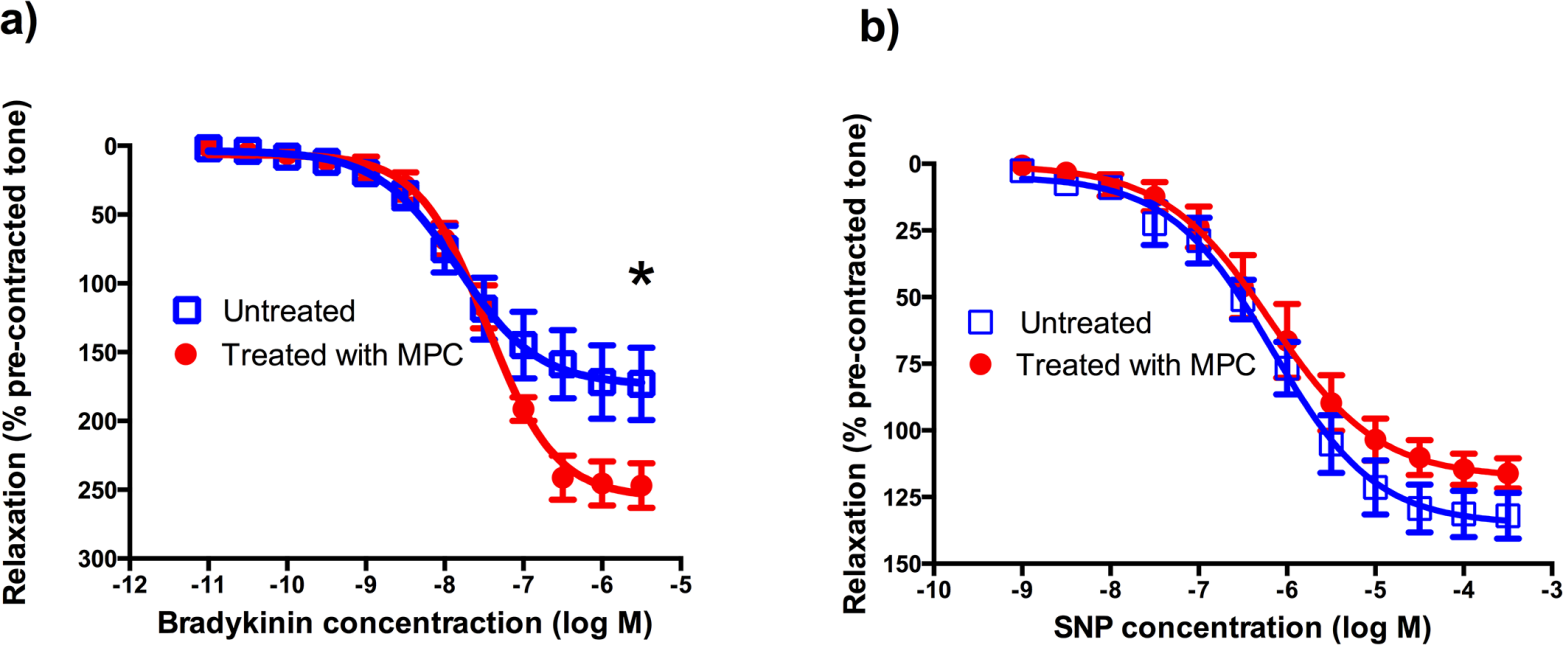
Comparison of vasorelaxation in ovine coronary arteries in arthritic sheep treated and untreated with mesenchymal precursor cells (MPC). Arterial rings were contracted with endothelin-1, and relaxation responses to cumulatively increasing concentrations of (A) Bradykinin (BK), (B) Sodium nitroprusside (SNP), were expressed as a percentage relaxation of pre-contracted tone. Each point represents the mean ±SEM from 8 animals. * Indicates statistically significant difference (p < 0.05) in the maximum response for the dilation to bradykinin between treated and untreated animals (see Table 1).

Coronary arteries from arthritic sheep treated allogeneically with 150 million MPC intravenously showed a significant increase in their maximal response to the endothelium-dependent vasodilator bradykinin (253.6 ± 17.1% of pre-contracted tone vs 182.3 ± 27.3% for treated and untreated sheep respectively, p = 0.044, Table 1, Fig 3). There was no significant difference in the maximal response of the coronary arteries to the endothelium independent dilator SNP between the treated and untreated groups.

**Table 1:** EC_50_ values and maximum responses derived from arthritic sheep coronary artery responses, showing the effect of mesenchymal precursor cell treatment on dilation caused by endothelium-dependent and-independent vasodilators.

Vasodilator	Group	n	EC_50_ value (Molar; mean ± SEM)	P value	Max response (% of pre-contracted tone;mean ± SEM)	P value	Hill slope (mean ± SEM)
Bradykinin	Treated	8	3.7 ± 0.9 x 10^–8^		253.6 ± 17.1		1.2 ± 0.1
	Untreated	8	2.9 ± 1.1 x 10^–8^	0.579	182.3 ± 27.3 *	0.044	1.1 ± 0.2
SNP	Treated	8	2.1 ± 1.2 x 10^–6^		122.6 ± 7.6		1.1 ± 0.1
	Untreated	8	8.4 ± 2.2 x 10^–7^	0.302	130.6 ± 7.9	0.475	0.8 ± 0.1

* Indicates significant difference between groups; unpaired *t*-test. SNP, sodium nitroprusside.

### Digital arteries

**Fig 4:**
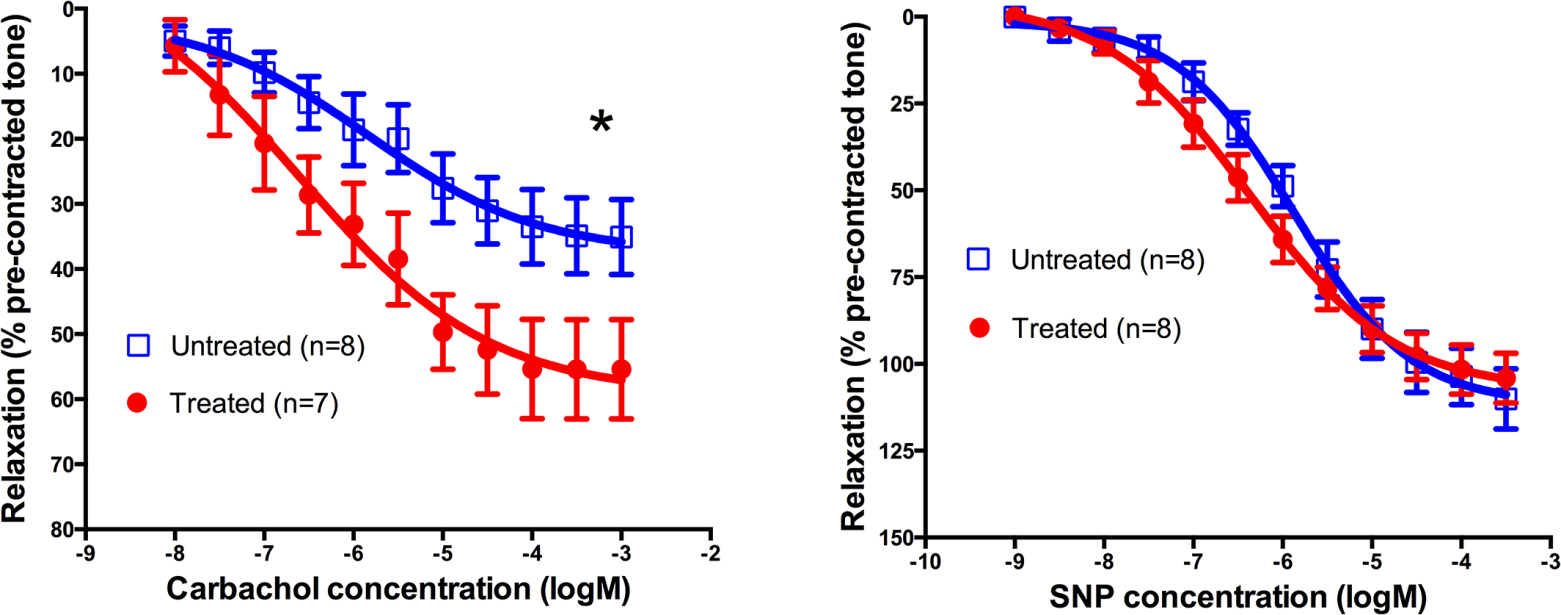
Comparison of vasorelaxation in ovine digital arteries in arthritic sheep treated and untreated with mesenchymal precursor cells (MPC). Arterial rings were contracted with 5-hydroxytryptamine (5-HT), and relaxation responses to cumulatively increasing concentrations of (A) Carbachol and (B) Sodium Nitroprusside (SNP) were expressed as a percentage relaxation of pre-contracted tone. Each point represents the mean ±SEM from 7–8 animals. * Indicates statistically significant difference (p < 0.05) in the maximum response for the dilation to carbachol between treated and untreated animals (See Table 2).

Digital arteries from arthritic sheep treated allogeneically with 150 million MPC intravenously showed a significant increase in their maximal response to the endothelium-dependent vasodilator carbachol (57.0 ± 7.9% of pre-contracted tone vs. 35.6 ± 5.2% for treated and untreated sheep respectively; p = 0.037; Table 2 and Fig 4). Carbachol data from one sheep in the treated group was excluded due to damage during dissection. There was no significant difference in the maximal response of the digital arteries to the endothelium independent dilator SNP between the treated and untreated groups.

**Table 2:** EC_50_ values and maximum responses derived from arthritic sheep digital artery responses, showing the effect of mesenchymal precursor cell treatment on dilation caused by endothelium-dependent and-independent vasodilators.

Vasodilator	Group	n	EC_50_ value (Molar; mean ± SEM)	P value	Max response (% of pre-contracted tone; mean ± SEM)	P value	Hill slope (mean ± SEM)
Carbachol	Treated	7	1.6 ± 0.8 x 10^–6^		57.0 ± 7.9		1.4 ± 0.6
	Untreated	8	3.7 ± 1.7 x 10^–6^	0.328	35.6 ± 5.2 *	0.037	1.4 ± 0.4
SNP	Treated	8	5.7 ± 1.9 x 10^–7^		97.8 ± 6.3		0.7 ± 0.1
	Untreated	8	1.7 ± 0.6 x 10^–6^	0.098	105.5 ± 8.0	0.461	0.8 ± 0.1

* Indicates significant difference between groups; unpaired *t*-test. SNP, sodium nitroprusside.

## Discussion

Trafficking studies have indicated that MSCs are able to home to sites of inflammation following systemic administration, however only a very small proportion of cells reach the inflamed target tissue [27,28,29]. It has therefore been postulated that following intravenous administration these cells may act systemically, by modulating the actions of recipient immune cells. It is therefore hoped that MPCs may have beneficial effects not only on local inflammation, but also the systemic sequelae related to inflammation in remote tissues.

This study evaluated the acute anti-inflammatory activities of immunoselected allogeneic STRO-3^+^ ovine Mesenchymal Precursor Cells (MPCs) in an ovine model of CIA. This collagen-induced arthritis model in the sheep causes initially moderate lameness, which steadily declines over a few days, and it is associated with an influx of inflammatory cells into the synovium of the affected hock joint [21]. The T cell subsets (CD4^+^, CD8^+^ and γδ T cells), B cells and particularly CD14^+^ monocytes/macrophages are all increased significantly in the arthritic synovial membrane of the collagen-injected joints compared to the contralateral right hock joints and those of the normal control sheep [21,23]. The present study demonstrated a systemic anti-inflammatory effect of MPCs following IV administration. In rodent CIA models, where systemic administration of MSCs has reduced disease severity, it has been associated with the suppression of T cell activation, a reduction in serum pro-inflammatory cytokine expression, and the induction of Foxp3^+^ regulatory T cells with an immunosuppressive phenotype [30,31,32]. However, it remains unknown whether systemically administered MSCs (and MPCs) directly interact with T cell populations, or act indirectly via other cell populations and anti-inflammatory cytokines.

MPC treatment was associated with a marked increase in plasma IL-10 observed 24 hrs following IV administration of cells. IL-10 is a functional feedback regulator of immune responses that inhibits a wide range of inflammatory and immune responses, down regulates the expression of TH1 cytokines [33,34,35], inhibits TH2 and allergy responses [34], sustains the expansion of Treg cells [36,37] and is known to reduce the severity of RA [33]. It is reported that IL-10 achieves this by acting directly on the genes of pro-inflammatory cytokines at the level of transcription [38]. IL-10 is expressed by synovial mononuclear cells in RA [39], has potent anti-inflammatory effects on synovial fluid mononuclear cells derived from patients with RA [40] and IL-10 deficient mice have more severe responses to CIA [41]. IL-10 is produced by many immune cells and expressed by a number of immune cells, particularly monocytes and macrophages and to a lesser extent lymphocytes [42]. It may also be produced by MSC [43]. The current study did not address which cell types produced the peak of IL-10 in blood; although MPC themselves may have produced some of the IL-10, given such a high concentration it is more likely that MPC were inducing monocytes or macrophages to produce this cytokine [44].

IL-6 is a multifunctional cytokine with wide-ranging effects on immune and inflammatory processes [45]. IL-6 induces B cell differentiation and immunoglobulin production, acute phase protein synthesis, T cell activation and differentiation, and macrophage differentiation. This pro-inflammatory cytokine is a key stimulator of the acute inflammatory response through the production of acute phase proteins, but also plays a role in the transition from acute to chronic inflammatory responses [46]. During acute inflammation, leukocyte infiltration is dominated by neutrophils, however chronic inflammatory processes are associated with the infiltration of mononuclear cells such as macrophages. When IL-6 is complexed to its soluble receptor sIL-6Rα it activates endothelial cells to produce IL-8 and monocyte chemoattractant protein-1, which attract circulating monocytes [46,47]. This initiates the transition from neutrophil to monocyte recruitment at the site of inflammation. For this reason, IL-6 is thought to play a crucial role in both the initiation and persistence of chronic inflammatory diseases.

Following IA collagen administration (day 28), the levels of IL-6 rose rapidly in the blood in all sheep, but there was no significant difference in plasma IL-6 levels between treated and untreated sheep post treatment. While we hypothesised that plasma IL-6 levels might decrease in MPC treated sheep, we were unable to demonstrate this clearly in our model. Possible factors affecting the levels of IL-6 in blood include binding of IL-6 to sIL-6Rα, which is also implicated in the pathogenesis of RA [48,49], and other kinetic factors relating to production, removal and retention in blood [50]. Plasma TNF-α was also measured in this study, using ovine and bovine specific antibodies, however any spike in plasma levels may have been very short lived (<24h). Measured levels were close to the limit of detection in this study, and there were no significant differences detected between treatment groups.

In this study, plasma fibrinogen and serum amyloid-A were used as biomarkers of the systemic inflammatory response. These acute phase proteins have been well characterised in veterinary medicine, and have been utilised in other sheep inflammatory models [51,52,53]. In this study, sheep treated with 150 million MPC intravenously displayed a significant reduction in the magnitude of their fibrinogen level elevations following arthritis induction, but the therapy did not completely ameliorate the fibrinogen spike following induction. The arthritic sheep treated with MPC also experienced a significantly lower peak of serum amyloid-A in the circulation. Taken together, these results suggest that MPC administration attenuated the development of the acute systemic inflammatory response following arthritis induction.

Systemic inflammation also results in a series of structural changes in lipoproteins, producing a lipid profile known as the atherogenic lipid phenotype, which has a demonstrated link to increased risk of CV disease [54,55]. A number of studies have reported lipid alterations in RA patients; however in the largest such study to date, the only parameter reaching statistical significance was lowered HDL levels in RA patients [56]. HDL is generally thought to have a protective effect on endothelial function [57,58,59]. Therefore, reduced HDL levels in RA patients may be an underlying mechanism contributing to the development of endothelial dysfunction. In this study, arthritic sheep treated with MPC displayed significant attenuation of the decline in HDL experienced following arthritis induction. This difference was most significant at day 8 following arthritis induction, however HDL levels in treated sheep continued to decline through the course of the study and by day 14 following arthritis induction, there was no difference between treated and untreated sheep. When considered together with the other results in this study, this data suggests that the HDL decline observed in this model in the early period (first 10 days) following arthritis induction is likely to be driven by the acute systemic inflammatory response (which was significantly attenuated by MPC administration), however the continued decline in HDL beyond this period may be propagated through a different pathway, on which MPC administration had a lesser effect. Extended studies will be required to determine if MPC administration has a longer term effect on the lipid profile phenotype in this animal model.

In chronic inflammatory diseases such as rheumatoid arthritis, it is believed that systemic inflammation is a key modulator of their increased risk of cardiovascular diseases [12,60]. Endothelial dysfunction has been extensively characterised in human RA patients [61,62], as well as in animal models of RA [22,63,64,65,66,67]. Dysfunctional endothelial cells are the first detectable cardiovascular change, and are a key effector cell type involved in the development of atherosclerotic lesions that predispose patients to acute coronary events [20,54,68,69]. Endothelial dysfunction is both an initial pathological step and a biomarker of the risk of future clinical cardiovascular disease, and is therefore of interest in the assessment of current and future therapeutics for RA and other chronic inflammatory diseases.

Intravenous administration of MPC to arthritic sheep significantly attenuated the development of endothelial dysfunction of the coronary artery in this study. Coronary endothelial dysfunction has been strongly associated with the development of cardiovascular disease in humans; and a number of studies have reported that poor coronary endothelium-dependent vasodilator function is a predictor of future cardiovascular events [68,70,71]. Our previous studies established that the ovine collagen-induced arthritis model produces coronary endothelial dysfunction two weeks following arthritis induction [22]. In this study, the maximal response of coronary artery segments to bradykinin in sheep treated with MPC was significantly improved. We have previously reported that bradykinin is an endothelium-dependent vasodilator in sheep coronary arteries, which exerts its effect primarily through the induction of NO production in endothelial cells [22]. This result therefore suggests that the systemic effect of MPC administration has restored the production of NO by endothelial cells in response to bradykinin stimulation. Although this response is most likely to be due to increased endothelial nitric oxide synthase (eNOS) enzyme activity and/or expression, for logistical reasons the intracellular mechanisms could not be investigated in more detail (using cell signaling inhibitors) in this study. Other potential mechanisms may be alterations in the expression of bradykinin receptors, or increased bioavailability of NO in MPC treated sheep (due to reduced reactive oxygen species production). Because of the relatively modest numbers of cells administered in these experiments, the effect of MPCs on endothelial cells is likely to be indirect; via modulation of key immune cell function in this animal model.

MPC treatment also significantly attenuated the development of endothelial dysfunction of the digital artery in this study. Digital arteries were investigated in this study to provide an example of a peripheral artery. Peripheral vascular disease has also been reported in the RA patient population [72]. This additional data also suggests that the effect of MPC treatment on endothelial function is systemic. In this study, the digital arterial segments from those sheep treated with MPC showed a significantly increased maximal response to the dilator carbachol. Our previous sheep studies have shown that carbachol is an endothelium-dependent dilator in sheep digital arteries, which (like bradykinin) exerts its effect through the induction of NO production in endothelial cells [22]. In both digital and coronary arteries, arthritic sheep treated with MPC showed no alterations in their response to the endothelium-independent dilator, SNP. This result indicates that MPC administration had no effect on vascular smooth muscle function, and that the difference observed in response to the dilators bradykinin and carbachol is due to selective functional differences in endothelial cells.

We propose that both the increase in plasma IL-10 and the decrease in pro-inflammatory cytokines such as IL-6 and TNF-α are directly associated with the maintenance of the endothelial function in the MPC-treated sheep. Pro-inflammatory cytokines have been shown to cause reduced production of nitric oxide from endothelial cells by down-regulating the expression and activity of nitric oxide synthase (eNOS), which may involve activation of the transcription factor NFkB and also the generation of reactive oxygen species which uncouple the eNOS enzyme [73]. Interleukin-10 has been shown to protect and restore eNOS expression (in part by inhibiting NFkB activity) and therefore prevents the impairment in endothelium-dependent vasorelaxation caused by pro-inflammatory cytokines [73].

No previous studies have examined the effect of MSC or MPC administration on endothelial cell function. In this model, endothelial function can be seen as a surrogate marker of the overall level of systemic inflammation over the course of the study. Our study provides the first evidence that MPC are able to modulate the development of the early cardiovascular sequelae of low-grade systemic inflammation. Although the mechanisms by which the MPC achieved this alteration will be the subject of future investigations, together the results suggest that the improvements in endothelial function with MPC treatment are likely to have been achieved indirectly through the modulation of the systemic inflammatory response following arthritis induction.

The mechanisms by which MPC are able to modulate inflammation in this animal model remain unclear. Many reviews have described the profound immunomodulatory properties of these cells in the context of autoimmune and inflammatory diseases [3,74,75,76,77]. However, much still remains unknown about the behaviour of these cells in animal models of disease. Following intravenous administration, one possibility was that the MPC trafficked to the inflamed synovial joint and directly inactivated key pro-inflammatory cell types. Alternatively, the cells may primarily interact with circulating leukocytes and/or with a remote population of leukocytes to reduce activation and cytokine production; or MPCs may produce their own soluble mediators which circulate and have far-reaching anti-inflammatory actions.

Studies tracking labelled MSCs to various organs after systemic administration have shown that the great majority of cells are initially trapped within the capillary beds of the lung; however significant numbers of cells also traffic to the spleen and liver [27,29,78,79]. The spleen (more specifically the subcapsular red pulp) is a site of residence for a significant reservoir population of monocytes which are mobilised into the circulation during inflammation [80]. The marked spike in plasma IL-10 levels observed 24h after the IV administration of MPC in the present study could be explained by the activation of significant numbers of anti-inflammatory (‘M2’) monocytes, or perhaps the conversion of quiescent monocytes or pro-inflammatory monocytes (M1) into M2 cells. Many of the subsequent anti-inflammatory effects of MPC may be driven indirectly via the actions of anti-inflammatory monocytes and cytokines such as IL-10. Similarly, if significant numbers of MSCs traffic to the liver, they might interact with Kupffer cells (macophages) lining the liver sinusoids. Further studies should be directed towards better understanding the fate of intravenously administered stem cells and the mechanisms by which they exert their immunomodulatory effect.

In conclusion, this is the first study to report the capacity of MPC to attenuate systemic inflammation and endothelial dysfunction associated with an animal model of arthritis. This important finding suggests that MPC show significant promise in modulating not only local disease activity in chronic inflammation such as a poly or mono-arthritis, but also the systemic sequelae of the condition. Further pre-clinical and mechanistic studies of the pathways involved in MPC modulation of systemic inflammatory diseases will inform the development this novel anti-inflammatory therapy.
